# Clinical Outcomes of Stage IIIC Poor-Risk Testicular Germ Cell Tumors: A Retrospective Analysis From a Tertiary Care Center

**DOI:** 10.7759/cureus.108000

**Published:** 2026-04-29

**Authors:** Maryam Imran, Shehar Bano, Afshan Khanum, Syed Abdullah Javaid Bukhari

**Affiliations:** 1 Medical Oncology, Shaukat Khanum Memorial Cancer Hospital and Research Centre, Lahore, PAK; 2 Cancer Registry and Data Management, Shaukat Khanum Memorial Cancer Hospital and Research Centre, Lahore, PAK

**Keywords:** non-seminomatous germ cell tumors, poor risk, prognostic factors, stage3c, testicular germ cell tumors

## Abstract

Background

Testicular germ cell tumors (TGCTs) are the most common malignancy in young men. While highly chemosensitive with excellent cure rates in early stages, outcomes remain suboptimal in advanced disease. This study evaluated clinical outcomes of patients with stage IIIC poor-risk TGCTs, a subgroup with limited survival data.

Methods

A retrospective review was conducted of 31 consecutive patients with stage IIIC poor-risk TGCTs treated between May 2021 and November 2023 at a tertiary care center. Clinical, pathological, treatment, and outcome data were analyzed.

Results

Thirty-one patients with stage IIIC poor-risk non-seminomatous TGCTs were included. Six patients (19.4%) died during first-line chemotherapy. Among the 25 patients who completed therapy, nine (36.0%) developed relapse. Mean progression-free survival (PFS) was 32.3 months, and mean overall survival (OS) was 31.1 months. Patients achieving tumor marker normalization after chemotherapy had markedly superior outcomes (mean PFS: 41.8 vs. 10.0 months; mean OS: 36.3 vs. 19.1 months). Brain metastases at diagnosis or on progression were associated with poor prognosis.

Conclusion

Patients presenting with poor performance status and visceral crisis are at high risk of early mortality during first-line chemotherapy. Tumor marker normalization strongly predicts improved survival, whereas persistent marker elevation and brain metastases are linked to unfavorable outcomes.

## Introduction

Germ cell tumors (GCTs) are a heterogeneous group of neoplasms originating from primordial germ cells, most commonly affecting the testes in young men. Testicular GCTs (TGCTs) account for approximately 1% of all male cancers but represent the most frequent malignancy among men aged 15-35 years [1].

Staging of testicular cancer is based on the American Joint Committee on Cancer (AJCC) TNM system, incorporating tumor extent and serum tumor markers [2]. The International Germ Cell Cancer Collaborative Group (IGCCCG) classification stratifies metastatic GCTs into good, intermediate, and poor-risk categories based on primary tumor site, metastatic burden, and serum tumor marker levels [3]. Patients with stage IIIC disease predominantly fall into the poor-risk category and experience aggressive disease progression and reduced survival rates [4,5].

Although cisplatin-based chemotherapy has markedly improved outcomes in metastatic disease, survival remains suboptimal in patients with poor-risk stage IIIC tumors [6].

The aim of this study was to evaluate progression-free survival (PFS) and overall survival (OS) in patients with stage IIIC poor-risk non-seminomatous GCTs and to assess the prognostic impact of tumor marker normalization in this population.

This study has not been previously presented or published in any form.

## Materials and methods

Study design and setting

This retrospective cohort study was conducted at the Department of Medical Oncology, Shaukat Khanum Memorial Cancer Hospital and Research Centre (SKMCH&RC), Lahore, Pakistan, a tertiary cancer center.

Participants

Consecutive male patients aged 18-45 years with histologically confirmed non-seminomatous testicular germ cell tumors (NSGCTs) treated between May 2021 and November 2023 were screened. Patients were included if they had stage IIIC disease and were classified as poor risk according to the International Germ Cell Cancer Collaborative Group (IGCCCG) criteria [3]. Patients with incomplete medical records precluding outcome assessment or those previously treated for another malignancy were excluded.

Data collection and variables

Data were extracted from electronic medical records using a standardized data sheet. Collected variables included age, baseline World Health Organization (WHO) performance status, metastatic sites (including brain involvement), baseline serum tumor markers (alpha-fetoprotein (AFP), beta-human chorionic gonadotropin (β-hCG), and lactate dehydrogenase (LDH)), first-line chemotherapy regimen, treatment completion status, relapse, and survival outcomes. Data extraction was independently verified by two investigators to minimize transcription errors.

Definitions

Tumor marker normalization was defined as normalization of AFP, β-hCG, and LDH within four to eight weeks after completion of first-line chemotherapy.

Relapse was defined as biochemical and/or radiologic progression after completion of first-line therapy.

Early mortality during chemotherapy was defined as death occurring during administration of first-line chemotherapy.

Brain metastasis was recorded if present at diagnosis or documented on follow-up imaging at progression.

Treatment

Patients received platinum-based chemotherapy (bleomycin, etoposide, and cisplatin (BEP); etoposide, ifosfamide, and cisplatin (VIP); or etoposide plus cisplatin (EP)) based on clinician judgment, baseline clinical status, and anticipated tolerance. Supportive care, dose adjustments, and hospitalization decisions were individualized according to clinical need.

Outcomes

The primary outcomes were PFS and OS. PFS was calculated from initiation of first-line chemotherapy to documented biochemical and/or radiologic progression. OS was calculated from initiation of first-line chemotherapy to death from any cause or last follow-up. Patients without an event were censored at the last clinical contact. Follow-up assessments included clinical evaluation, serum tumor markers, and imaging as per routine institutional practice.

Statistical analysis

Categorical variables were summarized as frequencies and percentages, and continuous variables as mean ± standard deviation (SD). Survival outcomes were estimated using the Kaplan-Meier method and compared using the log-rank test. A p-value < 0.05 was considered statistically significant. Statistical analysis was performed using Statistical Product and Service Solutions (SPSS, version 26.0; IBM SPSS Statistics for Windows, Armonk, NY). Survival curves were generated using the Kaplan-Meier method with 95% confidence intervals calculated using Greenwood's formula. Hazard ratios are calculated, but they require cautious interpretation due to the small sample size. Missing data were handled by case-wise deletion. Patients without events were censored at the last follow-up.

Ethics

The study was approved by the Institutional Review Board (approval number: EX-18-03-25-01), with waiver of informed consent due to its retrospective design.

## Results

A total of 31 consecutive patients with stage IIIC poor-risk NSGCT were included in this retrospective analysis. Baseline clinical characteristics demonstrated that the cohort largely comprised young adults, with a mean age of 31 years. In accordance with established treatment guidelines, BEP was administered as first-line chemotherapy in the majority of patients, followed by VIP, while EP was used in selected cases. Detailed baseline characteristics and treatment distribution are summarized in Table 1.

**Table 1:** Baseline clinical and treatment characteristics (n = 31) BEP = Bleomycin, Etoposide, Cisplatin; VIP = Etoposide, Ifosfamide, Cisplatin; EP = Etoposide, Cisplatin; IGCCCG = International Germ Cell Cancer Collaborative Group; SD = Standard Deviation

Variable	Category	n (%)
Age (years)	Mean+/-SD	31.0 +/-5.3
TNM Stage	IIIC	31 (100.0)
IGCCCG Risk Group	Poor Risk	31 (100.0)
First-Line Chemotherapy	BEP	22 (71.0)
	VIP	8 (25.8)
	EP	1 (3.2)
Brain Metastasis	At diagnosis	1 (3.2)
	On progression	4 (12.9)

During first-line chemotherapy, six patients (19.4%) experienced early mortality. Subgroup analysis of early deaths demonstrated that poor baseline performance status (Eastern Cooperative Oncology Group (ECOG): ≥ 3) and presentation with visceral crisis were associated with a higher risk of mortality. Sepsis was the most frequently identified cause of death in this subgroup. This is summarized in Table 2.

**Table 2:** Treatment completion, early mortality, and causes of early death (n = 31) WHO PS = World Health Organization Performance Status

Variable	n	%
Treatment outcomes		
Completed first-line chemotherapy	25	80.6
Early death during chemotherapy	6	19.4
Clinical characteristics among early deaths (n = 6)		
WHO performance status 3-4	5	83.3
Visceral crisis	4	66.7
Causes of early death (n = 6)		
Sepsis	3	50
Platinum-refractory disease	1	16.7
Spontaneous tumor lysis syndrome	1	16.7
Progressive visceral disease with brain metastasis	1	16.7

Among the 25 patients who completed first-line chemotherapy, nine (36.0%) developed relapse. Tumor marker normalization within four to eight weeks occurred in 16 patients (64.0%), while nine patients (36.0%) had persistently elevated markers. Relapse occurred more frequently in patients with persistent marker elevation.

Survival outcomes were further stratified according to tumor marker normalization versus persistent elevation following treatment. The mean PFS for the overall cohort was 32.3 months (95% CI: 24.6-40.0), as shown in Table 3.

**Table 3:** Progression-free survival (PFS) analysis

Group	Mean PFS (months)	95% CI	Log-rank p-value
Overall cohort	32.3	24.6-40.0	-
Tumor markers normalized	41.8	35.1-48.5	0.001
Tumor markers elevated	10	6.0-14.0	-

**Figure 1:**
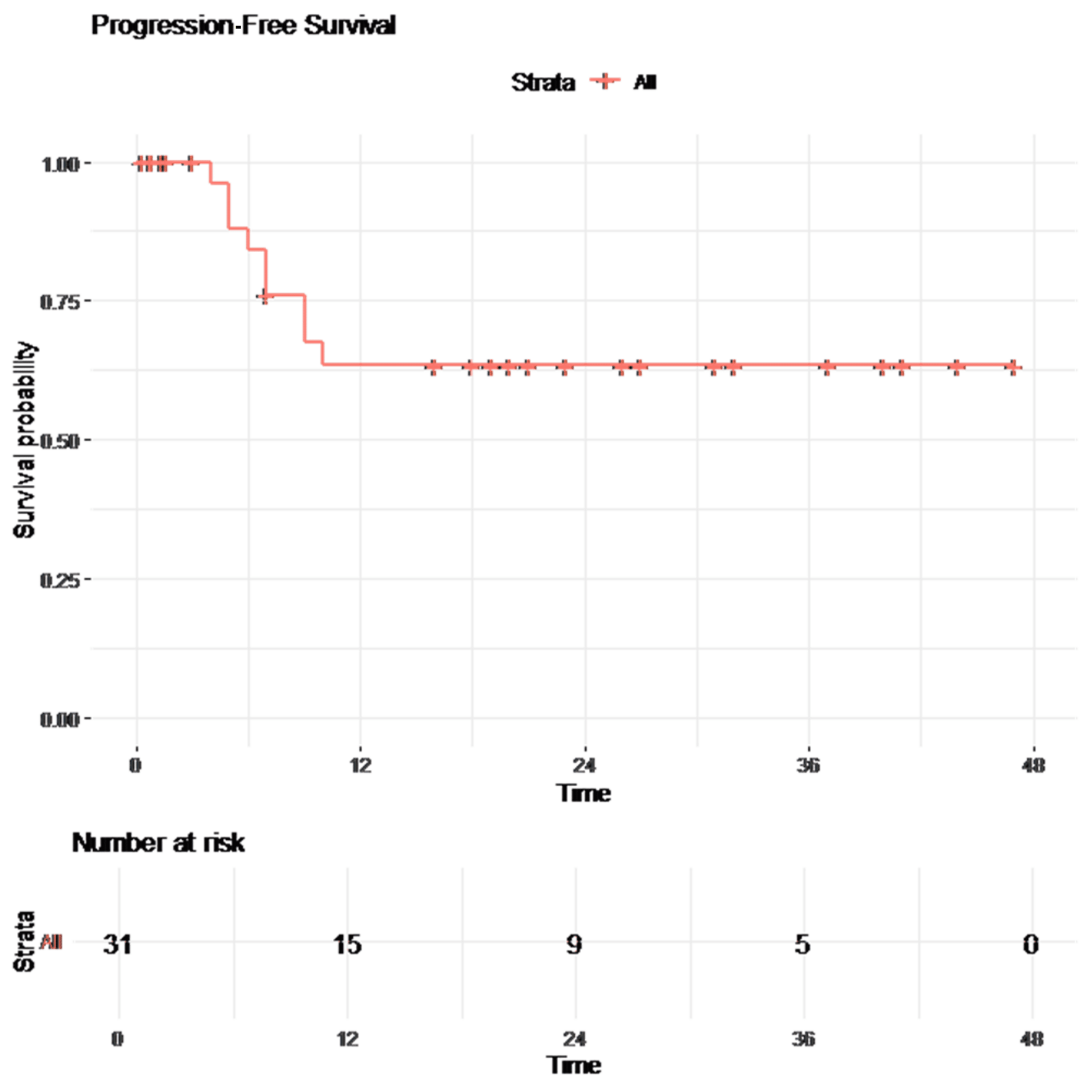
Kaplan-Meier curve for progression-free survival (PFS)

**Figure 2:**
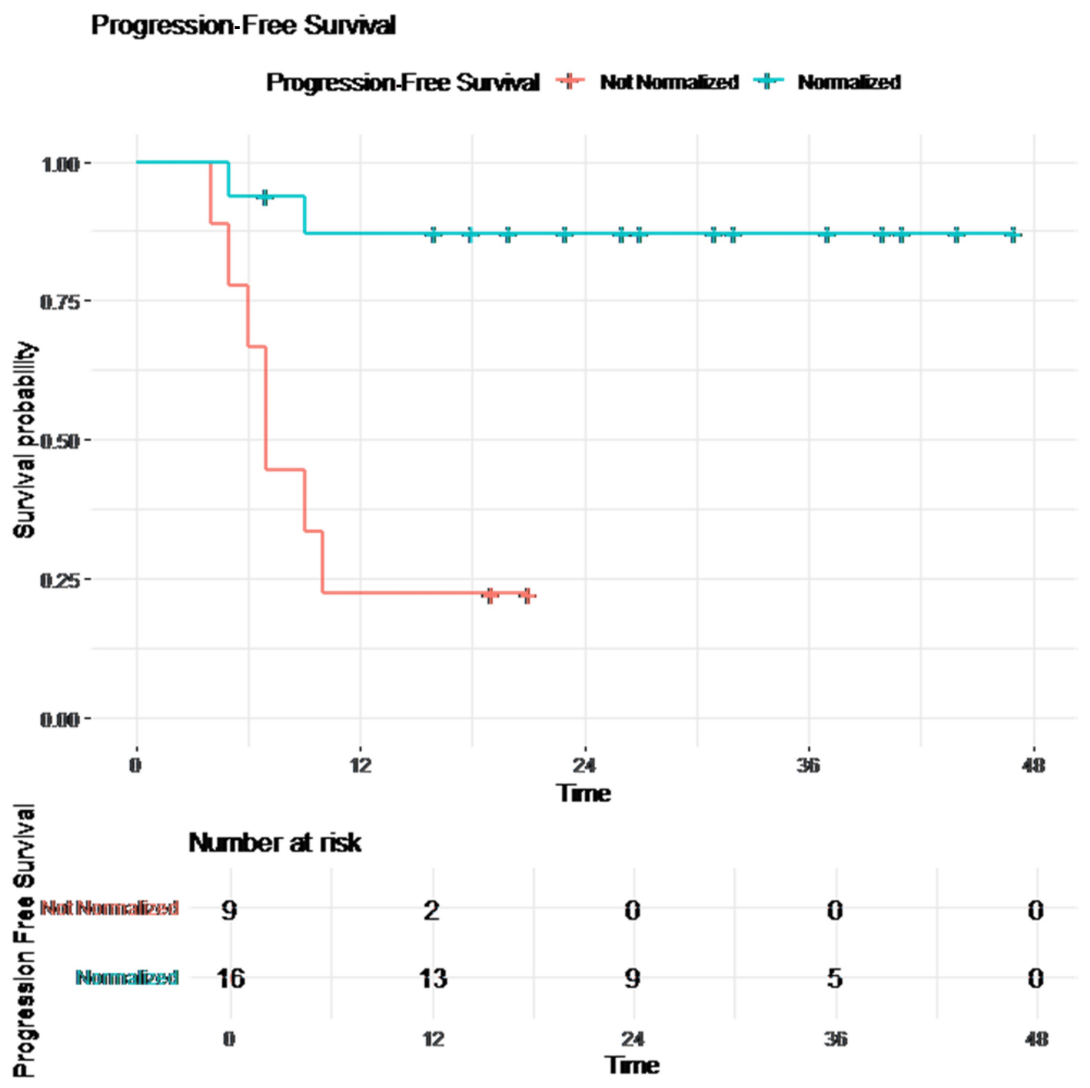
Progression-free survival (PFS) stratified by tumor marker normalization

Patients with normalized tumor markers demonstrated significantly improved PFS compared to those with persistent elevation (log-rank p = 0.001). Kaplan-Meier survival curves of PFS are presented in Figures 1-2.

The mean OS for the entire cohort was 31.1 months (95% CI: 25.8-36.4). Patients with normalized tumor markers had superior OS (log-rank p = 0.007), as shown in Table 4.

**Table 4:** Overall survival (OS) analysis

Group	Mean OS (months)	95% CI	Log-rank p-value
Overall cohort	31.1	25.8-36.4	-
Tumor markers normalized	36.3	30.4-42.2	0.007
Tumor markers elevated	19.1	15.5-22.6	-

**Figure 3:**
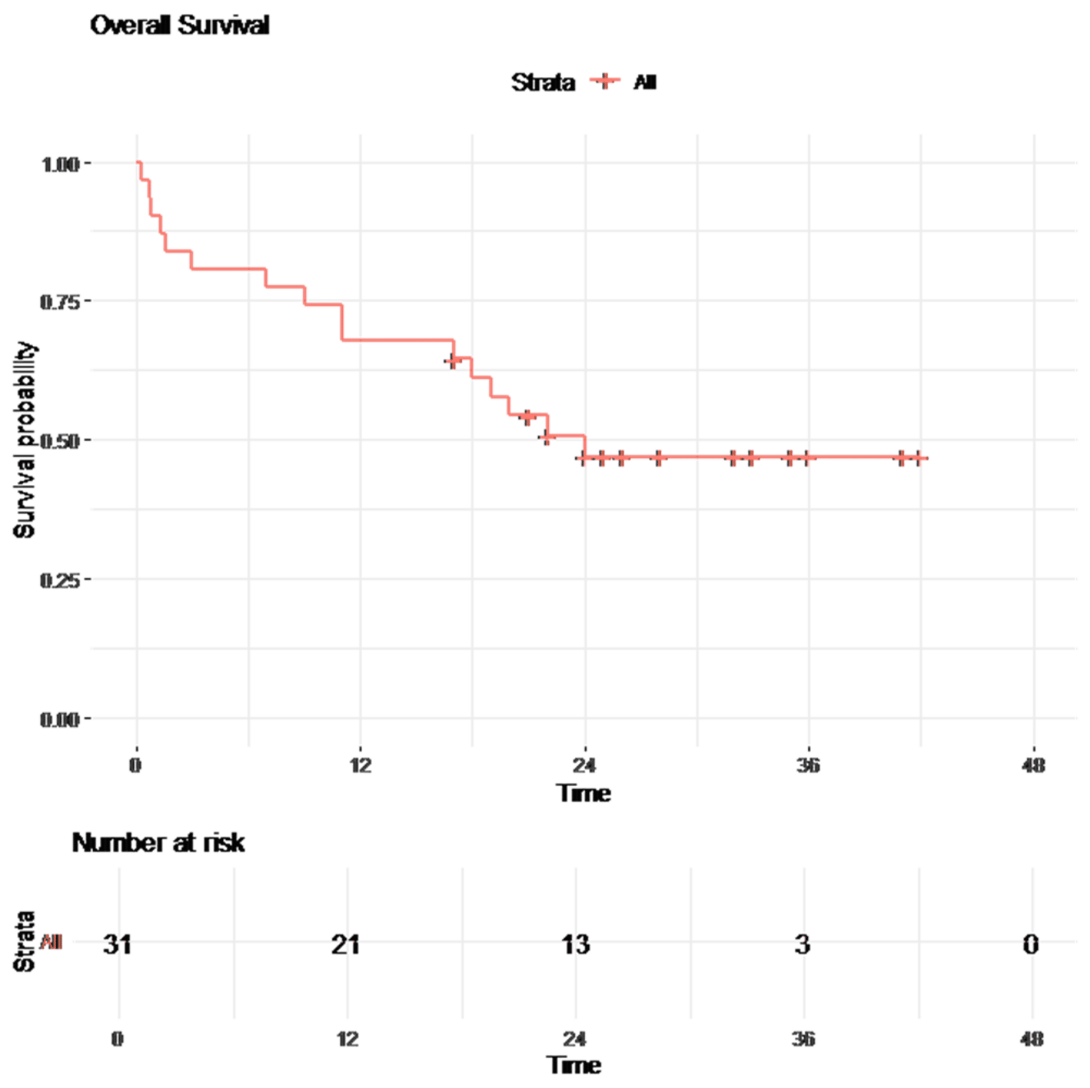
Kaplan-Meier curve for overall survival (OS)

**Figure 4:**
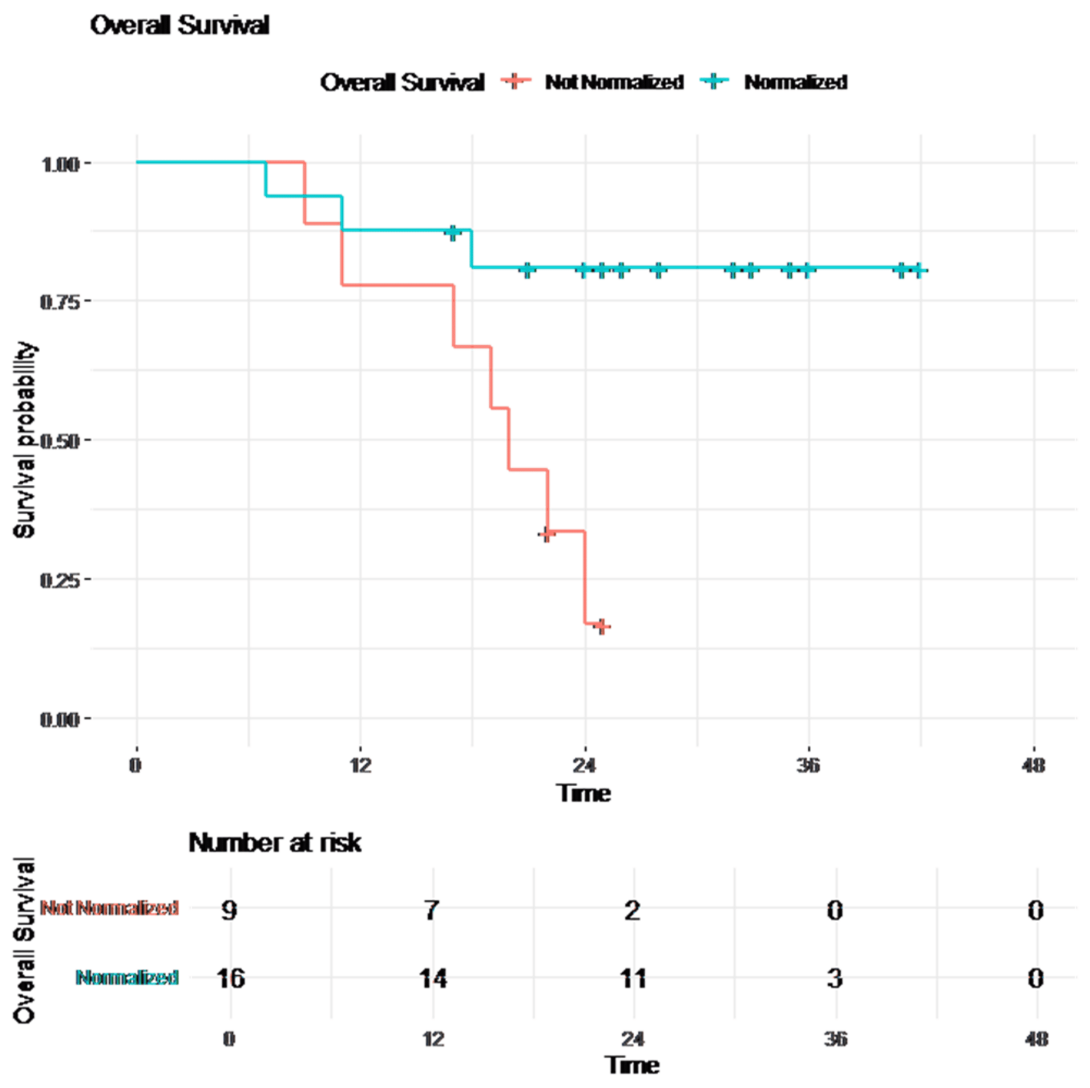
Overall survival (OS) stratified by tumor marker normalization

Kaplan-Meier survival curves of OS are presented in Figures 3-4.

PFS and OS were evaluated at 12, 24, and 36 months. The analysis showed that the majority of relapses and deaths occurred within the first 24 months after diagnosis. Beyond this period, the rate of disease progression and mortality appeared to stabilize, indicating that the early post-diagnosis phase is the most critical for adverse outcomes in this cohort. Survival estimates are reported with 95% confidence intervals. The relatively wider confidence intervals observed in the elevated tumor marker group likely reflect the small sample size and the limited number of patients remaining at risk at later time points. Time-specific survival rates are presented in Table 5.

**Table 5:** Time-specific survival rates NE: not estimated; OS: overall survival; PFS: progression-free survival

Time Point	PFS - Normalized (%) (95% CI)	PFS - Elevated (%) (95% CI)	OS - Normalized (%) (95% CI)	OS - Elevated (%) (95% CI)
12 months	100 (NE)	22.2 (6.5-75.4)	100 (NE)	100 (NE)
24 months	87.1 (71.8-100)	-	80.8 (63.4-100)	16.7 (3.2-88.2)
36 months	87.1 (71.8-100)	-	80.8 (63.4-100)	-

Cox proportional hazards regression analysis was performed to assess the impact of tumor marker normalization on survival outcomes. Hazard ratios (HRs) with corresponding 95% confidence intervals (CIs) were calculated. A total of 25 patients were included in this analysis.

Non-normalization of tumor markers was significantly associated with poorer overall survival. Patients with persistently elevated tumor markers had a more than fivefold higher risk of mortality compared to those whose markers normalized (HR: 5.34, 95% CI: 1.36-21.02, p=0.017).

A similar association was observed for PFS. Patients with non-normalized tumor markers had an approximately ninefold increased risk of disease progression or death (HR: 9.24, 95% CI: 1.90-45.03, p=0.006). These findings are summarized in Table 6.

**Table 6:** Cox proportional hazards analysis for survival outcomes

Outcome	Variable	HR	95% CI	p-value
Overall Survival (OS)	Tumor markers (not normalized vs normalized)	5.34	1.36-21.02	0.017
Progression-Free Survival (PFS)	Tumor markers (not normalized vs normalized)	9.24	1.90-45.03	0.006

## Discussion

Stage IIIC poor-risk NSGCTs represent one of the most aggressive subsets within germ cell malignancies. Although TGCTs are generally highly responsive to chemotherapy, patients classified as poor-risk continue to demonstrate significantly worse survival compared with good- and intermediate-risk groups. The original International Germ Cell Cancer Collaborative Group (IGCCCG) analysis reported five-year OS of approximately 48% in poor-risk patients [3], while subsequent contemporary analyses have shown modest improvements, with five-year OS ranging between 48% and 55% depending on metastatic burden and tumor marker levels [3,5].

In our cohort, 52% of patients died during follow-up, with a mean OS of 31 months. Although direct comparison with five-year OS data must be interpreted cautiously due to shorter follow-up, our findings are consistent with the persistently high mortality associated with stage IIIC disease in real-world clinical practice. Notably, early mortality during first-line chemotherapy occurred in 19% of patients, predominantly among those presenting with poor performance status and visceral crisis. Similar patterns of early death during induction chemotherapy have been described in large retrospective series, where poor ECOG/WHO performance status and high tumor burden were independent predictors of treatment-related mortality [7].

Brain metastases, although uncommon in germ cell tumors, are recognized as a major adverse prognostic factor in advanced disease. Central nervous system involvement has consistently been linked with inferior survival compared to patients without brain metastases [8].

Further contemporary analyses have refined prognostic stratification within poor-risk populations and confirmed the ongoing survival gap despite modern therapy [9]. Multidisciplinary management strategies are generally recommended in complex cases involving high tumor burden or central nervous system disease [10].

One of the most clinically meaningful findings of our study was the strong prognostic value of tumor marker normalization following first-line chemotherapy. Patients whose markers normalized within four to eight weeks demonstrated significantly superior PFS and OS compared to those with persistent elevation. This observation aligns with large international datasets demonstrating that post-chemotherapy tumor marker kinetics are independently associated with survival outcomes beyond baseline risk classification [9].

Reduction in serum tumor markers after chemotherapy likely reflects underlying tumor responsiveness and overall disease behavior, whereas persistent elevation may indicate residual viable tumor or platinum resistance. In relapsed or refractory settings, salvage approaches including conventional-dose and high-dose strategies have been evaluated, with variable outcomes across risk groups [11]. Earlier series focusing on germ cell tumor patients with brain metastases have also reported unfavorable survival, further underscoring the aggressive nature of central nervous system (CNS) involvement [12].

Additionally, patients presenting with visceral crisis - including extensive pulmonary or hepatic metastases - experienced higher rates of treatment-related complications and mortality. Large cohort analyses have similarly demonstrated that high-volume metastatic disease and compromised physiologic reserve significantly worsen prognosis in poor-risk populations [5,9]. These findings suggest that prognosis is determined not only by intrinsic tumor characteristics but also by the extent of disease and the patient's clinical condition at diagnosis.

Taken together, our results are concordant with established international data while providing contemporary real-world evidence from a tertiary care setting. Importantly, this study highlights that even in the era of cisplatin-based therapy, stage IIIC poor-risk NSGCT remains associated with substantial early mortality and relapse risk. Dynamic response markers, such as post-treatment tumor marker normalization, may provide additional prognostic refinement beyond static baseline risk categories and help guide individualized therapeutic strategies.

This study has several limitations, primarily related to the rarity of stage IIIC poor-risk non-seminomatous germ cell tumors and the resulting small cohort size; however, this reflects real-world incidence rather than selection bias. The retrospective single-center design may limit generalizability and introduce the possibility of incomplete data capture. Chemotherapy regimens were not completely uniform, as treatment decisions were individualized according to each patient's clinical condition. However, standard treatment protocols for this population include four cycles of BEP or, in cases with contraindications to bleomycin, four cycles of VIP, with comparable efficacy as per established guidelines. In selected high-risk symptomatic patients, an initial adapted cycle of cisplatin and etoposide may be used before proceeding to standard therapy. Therefore, subgroup analysis based on chemotherapy regimens was not considered essential in this cohort.

In addition, although follow-up was sufficient to evaluate early progression and mortality, longer observation is needed to better assess long-term survival trends. Multivariable Cox regression was not performed due to limited events, restricting adjustment for confounders. The relatively small sample size and limited number of events may affect the precision of the hazard ratio estimates, as reflected by the wide confidence intervals. Finally, molecular and genomic profiling was not routinely available, limiting deeper biologic stratification. Larger multicenter collaborations will be essential to further validate these findings in this rare but high-risk population.

## Conclusions

Stage IIIC poor-risk NSGCTs remain a therapeutic challenge, with substantial early mortality and relapse risk despite modern cisplatin-based chemotherapy. Early tumor marker normalization following first-line treatment appears to be a strong and clinically relevant prognostic indicator. In contrast, persistent tumor marker elevation, brain metastases, and visceral crisis are associated with a higher risk of adverse outcomes. Given the retrospective design and lack of multivariable adjustment, these findings should be interpreted as associations rather than definitive independent predictors. Multicenter prospective studies incorporating biologic and molecular stratification are needed to further validate these observations and optimize risk-adapted treatment strategies in this rare but aggressive subgroup.
